# Experimental demonstration of coherent superpositions in an ultrasonic pseudospin

**DOI:** 10.1038/s41598-019-50366-y

**Published:** 2019-10-02

**Authors:** Lazaro Calderin, M. Arif Hasan, Neil G. Jenkins, Trevor Lata, Pierre Lucas, Keith Runge, Pierre A. Deymier

**Affiliations:** Department of Materials Science and Engineering, University of Arizona, Tucson, Arizona 85721 USA

**Keywords:** Engineering, Materials science

## Abstract

We experimentally demonstrate the existence and control of coherent superpositions of elastic states in the direction of propagation of an ultrasonic pseudospin i.e., a *φ*-bit. The experimental realization of this mechanical pseudospin consists of an elastic aluminum rod serving as a waveguide sandwiched between two heavy steel plates. The Hertzian contact between the rod and the plates leads to restoring forces which couple the directions of propagation (forward and backward). This coupling generates the coherence of the superposition of elastic states. We also demonstrate *φ*-bit gate operations on the coherent superposition analogous to those used in quantum computing. In the case of a *φ*-bit, the coherent superposition of states in the direction of propagation are immune to wave function collapse upon measurement as they result from classical waves.

## Introduction

The quantum bit (qubit) is the critical component of future quantum information processing platforms^1^. A qubit is simply a physical system that supports a two-level quantum state, $|\psi \rangle =\alpha |0\rangle +\beta |1\rangle$ that can be formed as the coherent superposition of two pure states $|0\rangle$ and $|1\rangle$. For instance, a photon can be visualized as a qubit in polarization space. In this case, $|0\rangle$ and $|1\rangle$ represent the horizontal and vertical polarization, respectively. An electron spin is another physical realization of a qubit^2^, whereby $|0\rangle$ and $|1\rangle$ represent the up and down spin orientations. In the different forms of superconducting qubits, the states $|0\rangle$ and $|1\rangle$ are mapped onto discretized quantities characteristic of the relevant quantum Hamiltonian^3^. In addition to supporting coherent superpositions, what makes qubits so powerful for information processing is the ability by coupling multiple qubits of creating entangled i.e., non-separable states. This property confers exponential complexity to a *N*-qubit system which can then encode and ultimately process 2^*N*^ bits of information simultaneously.

The current quantum qubits, however, are based on quantum particles or quantum systems that easily lose their superposition of states in a noisy environment, or in large arrays, by decoherence. To increase coherence time, one then has to resort to operating the qubits at cumbersome cryogenic temperatures. Furthermore, measurement on quantum systems in superposition of states leads to collapse of the wave function onto pure states, requiring the use of statistical approaches to determining the original superposition. To overcome these critical drawbacks, one may call upon the notion of pseudospin which is a classical system that may exhibit properties isomorphic to true quantum spin systems. Pseudospin is playing a key role in understanding many fundamental quantum-like phenomena such as the anomalous quantum Hall effect. Unlike the electron spin, the pseudospin was traditionally considered as an unmeasurable quantity. Recently, however, it has been suggested that pseudospin is a real angular momentum that might manifest itself as an observable quantity. The concept of pseudospin, associated with Kramers degeneracy^4^, has recently been introduced in various topological systems. Besides the photonic^5,6^ and plasmonic systems^7^, pseudospin-dependent edge states in phononic systems (both acoustic^8,9^ and elastic^10–14^) have also been explored. Topological states for elastic waves have been predicted mostly in theoretical works^10–12^, and have only recently been experimentally demonstrated^13^. In ref.^13^, the authors experimentally demonstrated an elastic analog of the quantum spin Hall effects in a monolithically scalable configuration. Building on similar principles, we proposed theoretically, the concept of a one-dimensional elastic pseudospin, which we called a *φ*-bit. This elastic pseudospin is an elastic mechanical system in which the displacement field is describable by a wave function isomorphic to that of a quantum spin in that it possesses a spinorial character^15–18^. The *φ*-bit wave equation takes the form of a one-dimensional elastic wave equation with an additional term which then makes it isomorphic to the Klein-Gordon equation:

1 $$\frac{{\partial }^{2}u}{\partial {t}^{2}}-{\beta }^{2}\frac{{\partial }^{2}u}{\partial {x}^{2}}+{\alpha }^{2}u=0,$$

where *β* and *α* depend on the mass density of the elastic medium and elastic constants characteristic of the system being studied (vide infra).

It was previously shown^4^ that the modes of a *φ*-bit can be projected onto directions of propagation using a Dirac-like factorization:

2ab $$[{{\boldsymbol{\sigma }}}_{{\boldsymbol{x}}}\frac{\partial }{\partial t}+i\beta {{\boldsymbol{\sigma }}}_{{\boldsymbol{y}}}\frac{\partial }{\partial x}\pm i\alpha {\boldsymbol{I}}]\psi =0$$

where ***σ***_***x***_ and ***σ***_***y***_ are the 2 × 2 Pauli matrices and ***I*** is the 2 × 2 identity matrix. These modes, *ψ*, are expressible in terms of spinor amplitudes and orbital components. Furthermore, because of the ± sign, the complete set of states of the *φ*-bit includes non-dual solutions (“particle” and “antiparticle”). The non-dual solutions are plane waves: ${\psi }_{k}={c}_{0}{\xi }_{k}{e}^{(\pm )i{\omega }_{k}t}{e}^{(\pm )ikx}$ and ${\bar{\psi }}_{k}={c}_{0}{\bar{\xi }}_{k}{e}^{(\pm )i{\omega }_{k}t}{e}^{(\pm )ikx}$ where *ξ*_*k*_ and ${\bar{\xi }}_{k}$ are two by one spinors. The spinor amplitudes have the form $$(\begin{array}{c}{s}_{1}\sqrt{{\omega }_{k}\pm \beta k}\\ {s}_{2}\sqrt{{\omega }_{k}\mp \beta k}\end{array})$$ with *s*_1_ and *s*_2_ taking on the values +1 or −1 depending on the sign of *k* and *ω*_*k*_. Note that this spinor is solution of the continuous Dirac equation (Eq. 2(a,b)) and in a discrete representation of that equation, the spinor components have been shown to be complex^19^. The spinor solution corresponds to quasi-standing elastic waves with the components of the spinor representing the amplitude of the wave in the forward and backward directions of propagation, respectively. We can therefore define spin-like states in the direction of propagation of elastic states (forward $$|F\rangle =(\begin{array}{c}1\\ 0\end{array})$$ or $|0\rangle$ and backward $$|B\rangle =(\begin{array}{c}0\\ 1\end{array})$$ or $|1\rangle$) and crucially, coherent superposition of states: $({s}_{1}\sqrt{{\omega }_{k}\pm \beta k})|0\rangle +({s}_{2}\sqrt{{\omega }_{k}\mp \beta k})|1\rangle$. The superposition of states is tunable by frequency *ω* and/or wavenumber *k*. Because *s*_1_ and *s*_2_ can take values +1 and −1 we can realize the complete range of possible superpositions in the forward and backward space. These elastic waves with Dirac spinor characteristics have a half-integer spin analogue, i.e., a pseudospin. The superposition of states is coherent as the forward and backward components of the spinor are not independent of each other. It is not a classical mixed state or classical probabilistic mixture of forward and backward propagating elastic waves.

The elastic pseudospin superposition of states formed by a *φ*-bit can be stable at room temperature and decoherence free. It is measurable without wave function collapse as it represents an actual amplitude and not a probability amplitude. With these properties, the experimental realization of a *φ*-bit offers a transformative new solution to reach some of the goals of quantum information science using materials-based quantum analogues. In the present study, we physically realize an elastic system that exhibits pseudospin characteristics and we demonstrate coherent superposition of states in the directions of propagation. We also illustrate how one can manipulate the coherent states by tuning the wave frequency and wave number. This is equivalent to applying gate operations. Experimentally, the approach to realize a *φ*-bit is based on a piezo-actuated rod-like one-dimensional elastic waveguide subjected to nearly rigid boundary conditions on its external surface along its length. Elastic waves in the one-dimensional waveguide with free boundary conditions are described by the first two terms of Eq. (1). The rigidity condition introduces the third term of Eq. (1) and therefore the correlation between the directions of propagation. Here we introduce an experimental system which allows us to impose nearly rigid boundary conditions on the waveguide, and describe a simple theoretical model to characterize the expected general behavior of the system. We also report the results of the experimental measurements of the elastic states supported by an elastic rod waveguide with free boundary conditions and contrast it with those of the system with nearly rigid boundary conditions. Experimental observations with numerical calculations of elastic modes of relevant model systems using COMSOL simulations are discussed. Finally, we discuss how physical parameters such as frequency can be tuned to operate on the coherent elastic superposition of states. Conclusions are then drawn with respect to future development including the extension of the present work to multiple *φ*-bits and the creation of non-separable superpositions of states.

## Results

### Physical platform

**Figure 1:**
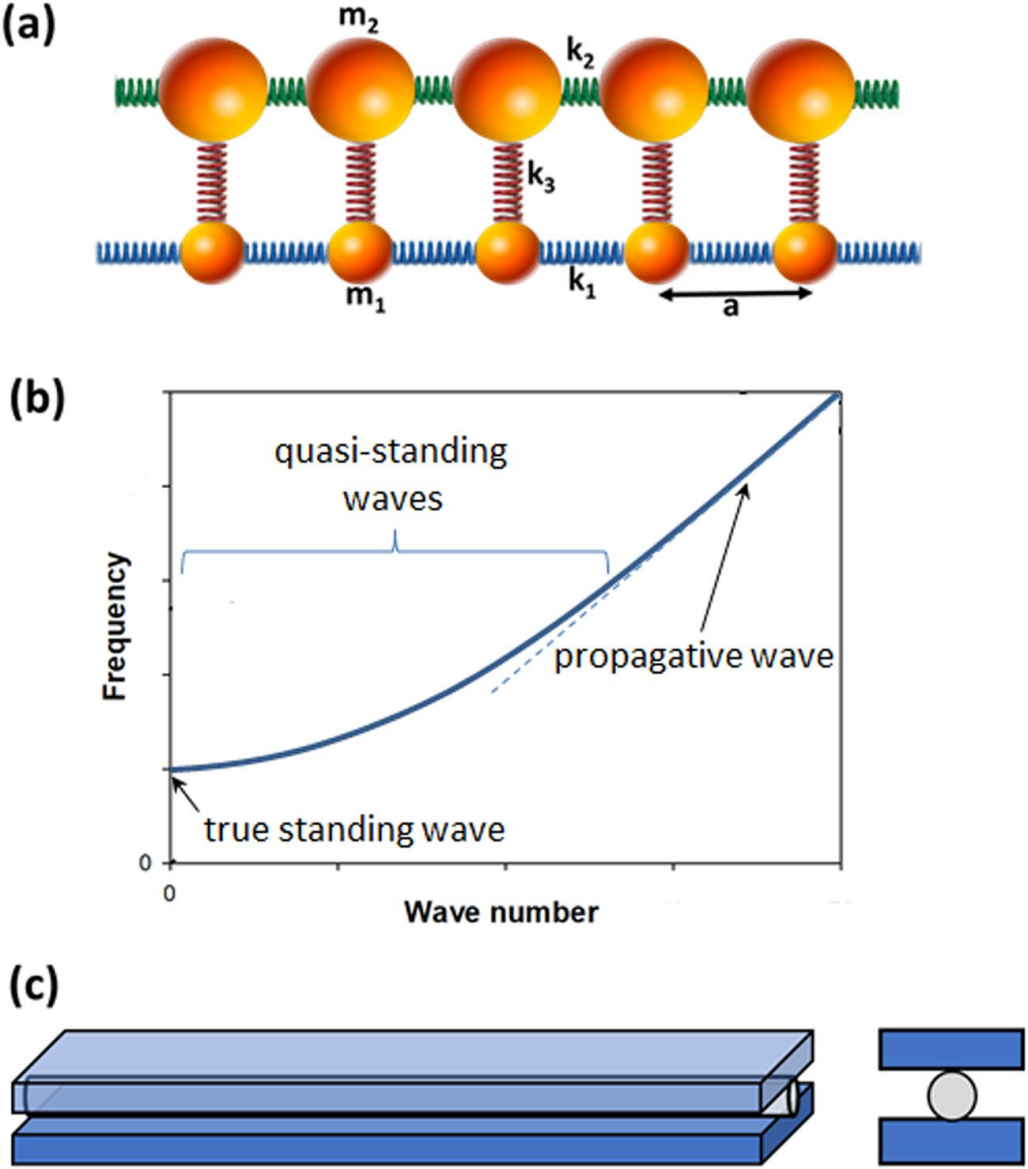
(**a**) Schematic illustration of the mass and spring model for a *φ*-bit system where two chains are coupled elastically. In the limit of masses *m*_2_ ≫ *m*_1_ the equation of motion for the chain composed of the lighter masses approaches that given by Eq. (1). The *k*_*i*_ with i = 1, 2, 3 are the stiffness of the springs. (**b**) Schematic illustration of the predicted *φ*-bit dispersion relation for the mass and spring system supporting coherent superposition of states as quasi-standing waves. (**c**) Side and front views of the physical platform for experimental realization of a *φ*-bit composed of an aluminum rod sandwiched between two steel plates. Pressure applied along the rod/steel plate sandwich establish elastic coupling through Hertzian contact. The large mass difference between the rod and the steel substrate provides the limiting condition for the rod waveguide to achieve the behavior of a *φ*-bit.

The experimental realization of a *φ*-bit requires a mechanical system which elastic wave behavior is effectively described by Eq. (1). The full experimental system is modeled as two mass and spring chains that are elastically coupled (Fig. 1a). The discrete elastic equations of motion are given by:

3a $${m}_{1}\frac{{\partial }^{2}{u}_{n}}{\partial {t}^{2}}-{k}_{1}({u}_{n+1}-2{u}_{n}+{u}_{n-1})-{k}_{3}({v}_{n}-{u}_{n})=0$$

3b $${m}_{2}\frac{{\partial }^{2}{v}_{n}}{\partial {t}^{2}}-{k}_{2}({v}_{n+1}-2{v}_{n}+{v}_{n-1})-{k}_{3}({u}_{n}-{v}_{n})=0$$

In Eq. (3), *u*_*n*_ is the *n*^*th*^ displacement of mass *m*_1_, *v*_*n*_ is the *n*^*th*^ displacement of mass *m*_2_, *k*_1_ (*k*_2_) is the stiffness of the springs coupling masses, *m*_1_(*m*_2_), *k*_3_ is the stiffness of the springs that couples masses *m*_1_ with *m*_2_ For the system considered here, the mass of the rod, *m*_1_, is much less than the mass of the remainder of the system, *m*_2_, so that we approach the equations of motion in Eq. (1). In the limit of long wavelength compared to the inter-mass spacing *a*, Eq. (3a,b) become:

4a $$\frac{{\partial }^{2}u}{\partial {t}^{2}}-{\beta }_{1}^{2}\frac{{\partial }^{2}u}{\partial {x}^{2}}-{\alpha }_{1}^{2}(v-u)=0$$

4b $$\frac{{\partial }^{2}v}{\partial {t}^{2}}-{\beta }_{2}^{2}\frac{{\partial }^{2}v}{\partial {x}^{2}}-{\alpha }_{2}^{2}(u-v)=0$$

where ${\beta }_{i}^{2}={k}_{i}{a}^{2}/{m}_{i}$ and ${\alpha }_{i}^{2}={k}_{3}/{m}_{i}$. Considering plane wave solutions $(u,v)=(A,B){e}^{i{\omega }_{k}t}{e}^{ikx}$, the eigen problem takes the form:

5a $$({\omega }_{k}^{2}-{\beta }_{1}^{2}{k}^{2}-{\alpha }_{1}^{2})A+{\alpha }_{1}^{2}B=0$$

5b $${\alpha }_{2}^{2}A+({\omega }_{k}^{2}-{\beta }_{2}^{2}{k}^{2}-{\alpha }_{2}^{2})B=0$$

The two dispersion relations of the system are found to be:

6 $${\omega }_{k}^{2}=\frac{b}{2}\pm \frac{1}{2}\sqrt{{b}^{2}-4c}$$

with $b=({\beta }_{1}^{2}+{\beta }_{2}^{2}){k}^{2}+{\alpha }_{1}^{2}+{\alpha }_{2}^{2}$ and $c={\beta }_{1}^{2}{\beta }_{2}^{2}{k}^{4}+({\beta }_{1}^{2}{\alpha }_{2}^{2}+{\beta }_{2}^{2}{\alpha }_{1}^{2}){k}^{2}$. The lower band (− of the ±) starts as the origin (${\omega }_{k=0}^{2}=0$) and the upper band (+ of the ±) possesses a cut off frequency ${\omega }_{k=0}^{2}={\alpha }_{1}^{2}+{\alpha }_{2}^{2}$.

Inserting Eq. (6) in Eq. (5a) gives a relation between the amplitudes of the eigen vectors:

7 $$\frac{B}{A}=-\,\frac{d}{2{\alpha }_{1}^{2}}\mp \frac{1}{2{\alpha }_{1}^{2}}\sqrt{{b}^{2}-4c}$$

where $d=({\beta }_{2}^{2}-{\beta }_{1}^{2}){k}^{2}+{\alpha }_{2}^{2}-{\alpha }_{2}^{2}$. Considering elastic springs with nearly identical stiffnesses, *k*_1_ = *k*_2_ = *k*_3_ (this approximation is justifiable because the current study involves metallic alloys with comparable moduli of elasticity), Eq. (7) reduces to:

8 $$\frac{B}{A}=-\,\frac{1}{2}(r-1)({a}^{2}{k}^{2}+1)\mp \frac{1}{2}\sqrt{{(r+1)}^{2}{({a}^{2}{k}^{2}+1)}^{2}-4r({a}^{4}{k}^{4}+2{a}^{2}{k}^{2})}$$

In this case the two chains only differ by their masses with the ratio $r=\frac{{m}_{1}}{{m}_{2}}$. If the two chains have identical masses, the lower band corresponds to a symmetric mode with $\frac{B}{A}=1$ and the upper band represents an antisymmetric mode with $\frac{B}{A}=-\,1$. We notice here that both chains possess identical amplitudes. In the limit of very disparate masses *m*_2_ ≫ *m*_1_, the slope of the lower band approaches zero and the second band approaches ${\omega }_{k}^{2}={\beta }_{1}^{2}{k}^{2}+{\alpha }_{1}^{2}$, the dispersion relation of a *φ*-bit. In that limit, the ratio of amplitudes *B*/*A* → 0 for the upper band and *B*/*A* → *a*^2^*k*^2^ + 1 for the lower band. The chain with the light mass (*m*_1_) supports modes in the lower band with very low amplitude compared to the heavier one. In the upper band, the lighter chain support modes with highest amplitude. In that limit, the set of equations (4) reduces to the single Klein-Gordon equation: $\frac{{\partial }^{2}u}{\partial {t}^{2}}-{\beta }_{1}^{2}\frac{{\partial }^{2}u}{\partial {x}^{2}}+{\alpha }_{1}^{2}u=0$, which is effectively Eq. (1).

A schematic of the resulting *φ*-bit dispersion relation is depicted in Fig. 1b. The true standing wave is composed of 50% forward/50% backward components while the propagative wave is 100% forward. Coherent superposition of states is then possible in the *φ*-bit band as quasi-standing wave composed of tunable fraction of forward and backward components.

This simple analytical model suggests that we can realize a *φ*-bit by elastically coupling two subsystems. The first subsystem is a light one-dimensional elastic waveguide and the second subsystem is composed of a massive elastic substrate. In practice, we use aluminum alloy rods sandwiched along their length between heavy steel plates (Fig. 1c). The Hertzian contact between the rod and the plates generate the side springs coupling *k*_3_ present in the discrete mass spring model. The *k*_2_ spring constant is given essentially by the rod Young’s modulus. An explicit connection between the properties of the experimental system and the discrete model is found as follows. The plates immobilize the lines of Hertzian contact so the system is effectively a rod with two fixed boundary lines. In the one-dimensional limit and considering only the longitudinal motion we get an elastic line coupled to a fixed line. Notice that independently of the number of Hertzian contact longitudinal lines and how they are distributed we get very similar equations in the limit of the infinite mass of the side chains; the only difference being in the *α*^2^. In the case of the three chains, for example, *α*^2^ is twice that of the two chains. The next step is to divide into equal longitudinal sections of length *a*, each with mass *m* = *ρA*_*s*_*a*, where *ρ* is the density of the rod and *A*_*s*_ the area of its cross-section. Finally, the sections are reduced to points of mass *m* separated by the distance *a* and the spring constants are given by *k*_1_ = *β*^2^*m*/*a*^2^ and *k*_3_ = *α*^2^/*m*, where the *α* and *β* are determined experimentally, and then *k*_1_ and *k*_3_ could be estimated.

Details of the experimental system are outline in the Materials and Methods section.

### Experimental measurements

**Figure 2:**
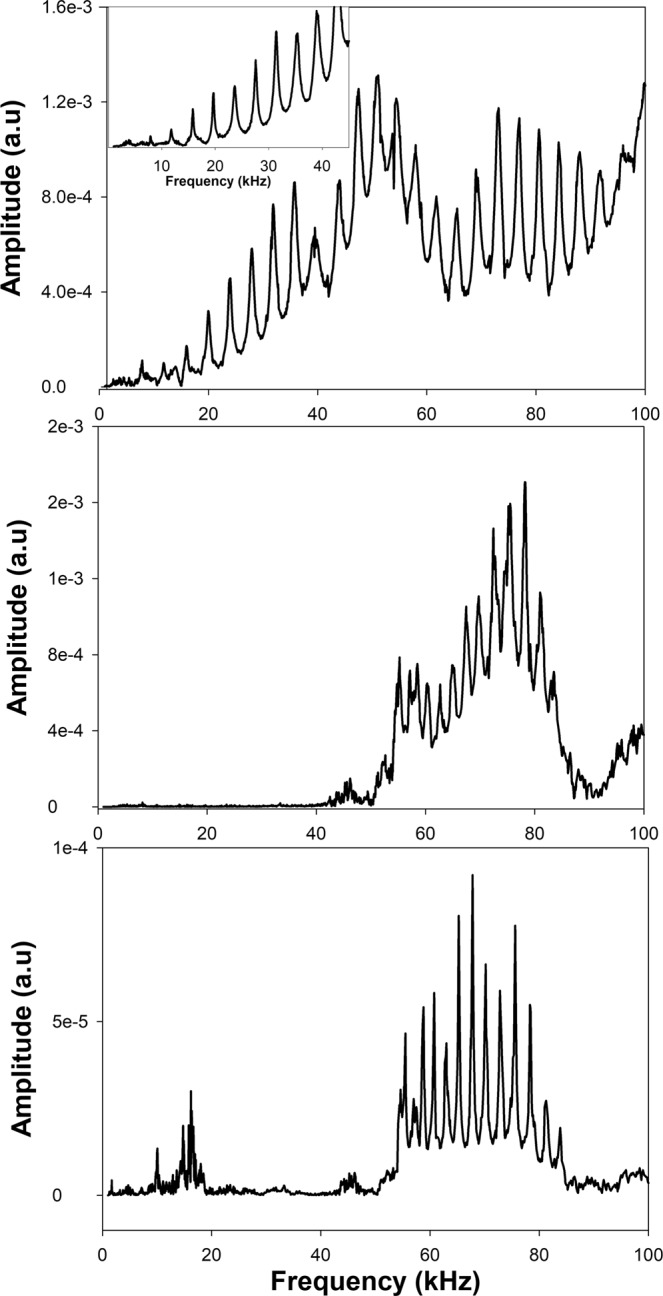
Transmission spectrum of the (**a**) free standing aluminum rod with rubber band attachment of the transducers and honey couplant (the inset is for transducers attached to the ends of the rod using lens mounts), (**b**) aluminum rod sandwiched between steel plates with gel couplant and lens mount attachments, and (**c**) same as (**b**) with kukui oil couplant with rubber band attachment. The transmission amplitude is in arbitrary units.

We investigate the behavior of an aluminum rod of length *L* = 0.6096 m with a density of 2,660 kg/m^3^. This density is significantly smaller than that of the steel substrate. Figure 2 reports the transmission spectra for the free standing aluminum rod and the rod sandwiched between the steel plates. We use different types of attachments of the transducers to the rod and ultrasonic couplants to illustrate the different types of behaviors that can be observed. However, we will show that the *φ*-bit modes are invariant and the couplant and attachment method only impact the number of modes that can be resolved.

**Figure 3:**
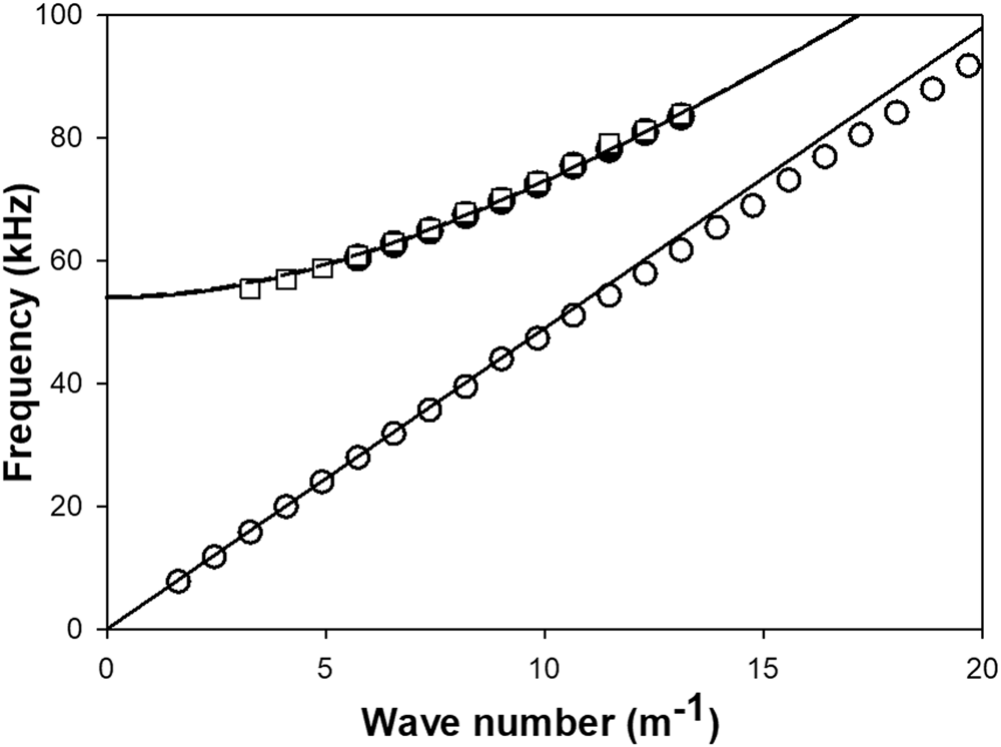
Dispersion relations for the aluminum rod and *φ*-bit determined and calculated from Fig. 2. The open circles are obtained from the resonances of the free standing aluminum rod. The solid line associated with the open circles is a fit to the low frequency resonances. The closed circles correspond to 10 resonant modes associated with the *φ*-bit modes of Fig. 2(b). The solid *φ*-bit band is a fit to these 10 modes using the *φ*-bit dispersion relation. The open squares represent 13 *φ*-bit modes identified in Fig. 2(c). The dashed line is a fit to these 13 modes using the *φ*-bit dispersion relation.

Figure 2(a) shows the transmission spectrum of the free standing rod using two types of attachment (rubber band for the main spectrum and lens mounts for the inset). The rubber band appears to impact the quality of the spectrum in the low frequency regime. The spectrum shows well defined resonances corresponding to the standing wave modes supported by this finite length rod. The wavelength corresponding to these standing waves can be easily determined from $\lambda =\frac{2L}{n}$ where *n* is an integer. Here it is straightforward to assign a value of *n* to each mode as they span the complete range of frequencies and their frequency separation is almost uniform. A wave number can subsequently be calculated as $k=\frac{1}{\lambda }$. It is therefore possible to plot the dispersion relation for the free standing rod by combining data from spectra of Fig. 2a (see Fig. 3). Note that this wave number is not an angular wave number which would be multiplied by 2*π*. The speed of sound of the nearly one-dimensional rod is extracted by fitting the low frequency resonant modes to a linear relation passing through the origin, that is a dispersion relation *ν* = *βk*. Here, we do not use angular frequencies but only frequencies which are related by a factor of 2*π*. We find that *β* = 4,900 m/sec. In Fig. 3, we see that as frequency increases beyond 60 kHz, the resonant modes of the free standing rod begin to deviate from the linear dispersion relation indicating that for short wavelengths the rod with finite cross section does not behave exactly like a one-dimensional elastic waveguide.

The rod sandwiched between the steel plates exhibits a transmission spectrum that is very different from the free standing rod (Fig. 2(b,c)). Both transmission spectra in Fig. 2(b,c) show a very large depression in the transmission amplitude below 50 kHz. The rubber band attachment appears to introduce some low frequency modes in Fig. 2(c). Beyond 20 kHz, the transmission amplitude along the rod is back to very small values. The very low transmission amplitude in the low frequency region of both transmission spectra is reminiscent of and consistent with the very low ratio of amplitudes, $\frac{B}{A}$, for elastic waves in the lower band propagating in the two chain model in the limit of a small ratio of masses, $r=\frac{{m}_{1}}{{m}_{2}}$. Above 50 kHz, the transmission spectrum shows well defined resonances with non-uniform frequency spacing. The resonances appear to be spaced more closely at low frequency. Therefore, the spectra show a passing band with well-defined resonances that are assigned to the *φ*-bit band (upper band of the two chain system).

To calculate a dispersion relation that would approach that of a *φ*-bit, we need to determine the wave number associated with the high frequency resonances observed in the spectra. This is done by selecting clear resonances of the rod below 80 kHz to avoid the loss of one-dimensionality above that frequency. The wave number of these resonances ought to be a multiple of 2 *L* since the finite length rod can only support standing waves. We label each resonance with the lowest frequency as being *n* = 1. We then calculate for each resonance “*n*” a cut-off frequency

9 $${\alpha }_{n}=\sqrt{{\nu }_{n}^{2}-{\beta }^{2}\Delta {k}^{2}{({n}_{0}+n)}^{2}}$$

where *υ*_*n*_ is the frequency of the resonance *n*, Δ*k* = 1/2*L* and *β* = 4,900 m/sec. We seek the integer value *n*_0_ which results in *α*_*n*_ with the least variance in “*n*”. We then determine the cutoff frequency *α* as the average over the *n*’s of all the *α*_*n*_. We use 10 resonances from Fig. 2(b) between 60 and 84 kHz and 13 resonances between 55 and 84 kHz from Fig. 2(c) to reconstruct the *φ*-bit band and determine the cutoff frequency. In the first case, we find *n*_0_ = 6 and a cutoff frequency, *α* = 53.960 kHz (standard deviation of 0.358 kHz). With the second set of data, we find *n*_0_ = 3 and a cutoff frequency, *α* = 54.320 kHz (standard deviation of 0.6532 kHz). The cutoff frequencies appear to be consistent with the measured transmission spectra where we observe a very low transmission. The cutoff frequencies are in excellent agreement with each other.

Figure 3 reports the different dispersion relations extracted from the transmission spectra. Analysis of the two transmission spectra of Fig. 2(b,c) give *φ*-bit dispersion relations in excellent agreement with each other. The experimental conditions of Fig. 2(c) enable us to resolve resonances with frequencies very close to the cutoff frequency giving exquisite control on the spinor states of the elastic waves.

We have used COMSOL to model the behavior of a free standing aluminum rod and an aluminum rod with two rigid line boundary conditions along its length at opposite sides around the perimeter of its cross section. The finite element grid and fixed boundary lines, used to model the *φ*-bit, are detailed in the Supplemental Text. The length of the rod is again *L* = 0.6096 m. The eigen problem for the elastic wave field equation is solved in real 3D and frequency space, the longitudinal modes are identified by the mode shapes and their wave number estimated by the formula *k* = (*N* − 1)/(*2L*), with *N* being the number of nodes. Some of the longitudinal modes hybridize with other types of modes making their identification from mode shapes impossible and therefore they are omitted. Other than the length and diameter of the rod, three physical parameters are needed in the calculation: the density, Poisson ratio and Young’s modulus. We use the experimentally determined density and we fix the value of Poisson ratio to 0.33. The Young’s modulus is obtained ensuring that the frequency spacing between subsequent predicted longitudinal eigen modes of the free standing rod matches, within 100 Hz, the experimental value in the low frequency regime, namely 3,950 Hz. The optimized Young’s modulus E = 60 GPa. The same physical parameters are used for the *φ*-bit model.

**Figure 4:**
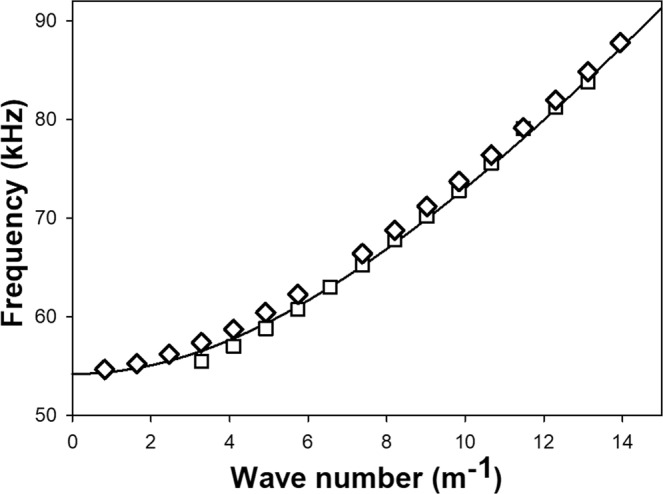
Experimental *φ*-bit dispersion relations for the aluminum/steel system determined and calculated from Fig. 2(c) and reported in Fig. 3 (solid line and open squares). Eigen modes calculated with COMSOL (open diamonds).

The *φ*-bit eigen values calculated with the COMSOL model are reported in Fig. 4 and compared with the experimental *φ*-bit resonances and fitted dispersion relation of the aluminum rod. The agreement between the numerical, theoretical and experimental eigen dispersion bands is remarkable. The numerical and experimental data show only a very modest difference of at most a few hundred Hz. This minor discrepancy might be due to the rigid nature of the boundary conditions along the rod in the numerical model which differs from (a) the elastic coupling associated with the Hertzian contact between the aluminum rod and the steel plates and (b) the finite ratio of the mass of the rod to that of the steel substrate. The rigid boundary condition likely overestimates the cutoff frequency. The agreement between experimental and numerical data strongly supports the *φ*-bit nature of the aluminum rod/steel plate assembly.

## Discussion

As noted in the introduction, plane wave solutions: ${\psi }_{k}={c}_{0}{\xi }_{k}{e}^{+i{\omega }_{k}t}{e}^{+ikx}$ of the Dirac-factored elastic wave equation of a solid *φ*-bit possesses a normalized two by one spinor amplitude:

10 $${\xi }_{k}=\frac{1}{\sqrt{2{\omega }_{k}}}(\begin{array}{c}\sqrt{{\omega }_{k}+\beta k}\\ \sqrt{{\omega }_{k}-\beta k}\end{array})=\frac{1}{{\sqrt{2\nu }}_{k}}(\begin{array}{c}\sqrt{{\nu }_{k}+\beta k}\\ \sqrt{{\nu }_{k}-\beta k}\end{array})$$

The second relation uses frequency instead of angular frequency, and wave number in m^−1^ instead of rad.m^−1^. This spinor corresponds to quasi-standing elastic waves with the components of the spinor representing the amplitude of the wave in the forward and backward directions of propagation, respectively. The spinor represents coherent superposition of states of the elastic waves as the amplitudes of the forward and backward components of the wave are not independent of each other. It is not possible to change the amplitude of the forward component without changing the backward component. This is quite different from a statistical superposition of states where there are no constraints on the choice of the amplitude of the superposed waves. Using the notation introduced earlier for pure states: forward $$|F\rangle =(\begin{array}{c}1\\ 0\end{array})$$ or $|0\rangle$ and backward $$|B\rangle =(\begin{array}{c}0\\ 1\end{array})$$ or $|1\rangle$, the coherent superposition of states in the *φ*-bit band can be written as:

11 $${\xi }_{k}=\frac{1}{\sqrt{2{\nu }_{k}}}(\sqrt{{\nu }_{k}+\beta k})|0\rangle +\frac{1}{\sqrt{2{\nu }_{k}}}(\sqrt{{\nu }_{k}-\beta k})|1\rangle ={\xi }_{F}({\nu }_{k},k)|0\rangle +{\xi }_{B}({\nu }_{k},k)|1\rangle $$

The superposition of states in the direction of propagation is tunable by frequency *ν*_*k*_ and/or wavenumber *k*. Changing frequency is therefore equivalent to applying a unitary transformation onto the spinor state. This point is illustrated by considering Table 1. In Table 1, we list the 13 experimental *φ*-bit modes of the aluminum rod and the corresponding normalized spinor amplitudes *ξ*_*F*_ and *ξ*_*B*_. We recall that we experimentally determined *β* = 4,900 m/sec. The experimental data reported in Table 1 span a reasonable interval of possible values of *ξ*_*F*_ and *ξ*_*B*_ which can range from $$(\begin{array}{c}0.7071\\ 0.7071\end{array})$$ at the cutoff frequency to $$(\begin{array}{c}1\\ 0\end{array})$$ in the limit of large frequencies. The coherent superpositions of states are quasi-standing waves and the percentages of the amplitude in the forward and backward directions are listed in the fifth column of the table. While the complete range of coherent states goes from 50% forward/50% backward to 100% forward, the experimental *φ*-bit allows us to manipulate a smaller but significant range of states between 64.5%/35.5% to 88.3%/11.7%. The first state is the closest to a true standing wave and the latter one is approaching a purely propagative wave.

**Table 1:** The first two columns are the experimental frequency and wave number of 13 *φ*-bit modes on the aluminum/steel assembly.

*ν* (kHz)	*k*(m^−1^)	*ξ*_*F*_	*ξ*_*B*_	%F/%B
55.50	3.2808	0.8030	0.5960	64.5/35.5
57.00	4.1010	0.8224	0.5690	67.6/32.4
58.80	4.9213	0.8397	0.5431	70.5/29.5
60.75	5.7415	0.8553	0.5181	73.2/26.8
63	6.5617	0.8690	0.4948	75.5/24.5
65.25	7.3819	0.8816	0.4720	77.7/22.3
67.80	8.2021	0.8924	0.4512	79.6/20.4
70.20	9.0223	0.9027	0.4303	81.5/18.5
72.80	9.8425	0.9117	0.4108	83.1/16.9
75.60	10.6627	0.9195	0.3930	84.6/15.4
79.10	11.4829	0.9250	0.3799	85.6/14.4
81.25	12.3031	0.9333	0.3592	87.1/12.9
83.85	13.1234	0.9399	0.3414	88.3/11.7

The third and fourth columns report the normalized forward and backward spinor amplitudes of the coherent superposition of states corresponding to the experimental modes, namely *ξ*_*F*_ and *ξ*_*B*_ (see Eq. (11) for details). Column 5 includes the squares of *ξ*_*F*_ and *ξ*_*B*_ which indicate the % of the quasi-standing waves which propagate in the forward direction and the backward direction, respectively.

Adjusting the frequency of the driving transducer can therefore be used to operate on the spinor state of the *φ*-bit as there is a one-to-one correspondence between resonant frequency and the value of the spinor. The action of changing the driving frequency is equivalent to a transformation of the spinor state. For instance, the transformation that takes the forward propagating state $$(\begin{array}{c}1\\ 0\end{array})$$ and transforms it into the standing wave state $$(\begin{array}{c}0.7071\\ 0.7071\end{array})$$ takes the form:

12 $$(\begin{array}{c}0.7071\\ 0.7071\end{array})=\frac{1}{\sqrt{2}}(\begin{array}{cc}1 & 1\\ 1 & -1\end{array})(\begin{array}{c}1\\ 0\end{array})$$

The transformation matrix $$H=\frac{1}{\sqrt{2}}(\begin{array}{cc}1 & 1\\ 1 & -\,1\end{array})$$ is the usual Hadamard transformation^20^ but is only one specific case of a general unitary transformation:

13 $$U(\gamma )=(\begin{array}{cc}\sqrt{\gamma } & \sqrt{1-\gamma }\\ \sqrt{1-\gamma } & -\sqrt{\gamma }\end{array})$$

The Hadamard gate is obtained for $\gamma =\frac{1}{2}$. We can now consider, for instance, the transformation realized physically by detuning the frequency of the driving transducer from 83.85 kHz to 55.50 kHz. This transformation is represented as: $$(\begin{array}{c}0.8030\\ 0.5960\end{array})=(\begin{array}{cc}\sqrt{\gamma ^{\prime} } & \sqrt{1-\gamma ^{\prime} }\\ \sqrt{1-\gamma ^{\prime} } & -\sqrt{\gamma ^{\prime} }\end{array})(\begin{array}{c}0.9399\\ 0.3414\end{array})$$ with *γ* ′ = 0.304. This is but one example of a single *φ*-bit gate operation. This type of gate operation is similar to the single qubit gate operation in quantum computing. In the case of spin-based qubits, one can transform a coherent superposition of up-spin and down-spin states by applying a laser pulse^21^. The challenge in quantum systems is that measurement on a quantum superposition of states leads to wave function collapse, that is, the state of the quantum system transforms from the superposition to a pure state. In the case of a *φ*-bit, the coherent superpositions of states in the direction of propagation are immune to wave function collapse as they result from classical waves.

**Figure 5:**
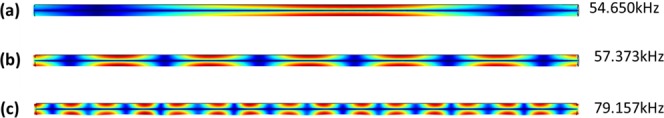
Square of the displacement field along the aluminum *φ*-bit model calculated using COMSOL. The figures correspond to coherent superposition of states (**a**) near the bottom of the *φ*-bit band, (**b**) in the region of large curvature of the band and (**c**) in the region of the band associated with nearly propagative modes. Red indicates large displacement and blue indicates small displacement.

We can use the COMSOL simulations to illustrate the displacement field associated with the coherent superposition of states. Figure 5(a) represents the *φ*-bit mode with the lowest resolvable frequency. One clearly sees in the figure the line along the length of the rod with rigid boundary conditions. This is a state very near the bottom of the *φ*-bit band. This mode is a quasi-standing wave with spinor $$(\begin{array}{c}0.7326\\ 0.6806\end{array})$$, which approaches the state of a standing wave which spinor amplitude is $$(\begin{array}{c}0.7071\\ 0.7071\end{array})$$. However, due to the fact that it may be visualized as the superposition of two waves traveling in opposite direction with slightly differing amplitudes (53.7% F/46.3% B), the displacement field will not be symmetrical about the middle of the rod as seen in the figure. As frequency increases this becomes less and less apparent. Figure 5(c) is characteristic of a nearly traveling wave with spinor $$(\begin{array}{c}0.9249\\ 0.3802\end{array})$$ and 85.5% forward character (14.5% backward). This quasi-standing wave appears as a standing wave in the figure because of the finite length of the rod. Figure 5(b) represents an intermediate superposition of states with spinor $$(\begin{array}{c}0.8192\\ 0.5735\end{array})$$ and 67.1% forward character.

## Conclusions

We have demonstrated experimentally the existence and control of coherent superpositions of elastic states in the direction of propagation of an ultrasonic pseudospin i.e., a *φ*-bit. The pseudospin states are supported by an elastic rod serving as a waveguide sandwiched along its length between massive plates. The Hertzian contact between the plates and the rod establishes restoring forces which result in quasi-standing wave elastic longitudinal modes that can be represented as spinors in the space of the direction of propagation along the waveguide. The experimental results are validated with theoretical and computational models. We show that by tuning the frequency of the resonances of the rod associated with the superpositions of states, one operates on the spinor state of the elastic waves. These operations are analogous to unitary operations commonly used to transform superposition of states in a true quantum spin qubit. Our work is one step toward implementing classical elastic systems which are analogous to quantum systems. These quantum analogues may exhibit the properties of quantum systems without some of the drawbacks associated with true quantum systems, such as wave function collapse and decoherence. While this paper focuses on a single *φ*-bit system analogous to single qubit, coupling *φ*-bits or coupling qubits is essential for implementing information processing platforms that can take on the exponential complexity associated with the non-separability of states in these coupled systems. We have addressed in other publications^19,22^ the theory of non-separability of elastic waves in coupled pseudospin systems. These theoretical studies revealed a rich variety of phenomena. For instance, in ref.^22^ we have demonstrated the possibility of achieving non-separability between different spinor and other degree of freedom in a system of elastically coupled waveguides. Moreover, in ref.^19^ we have shown the possibility of realizing non-separable spinor states in multiple coupled *φ*-bit systems.

## Materials and Methods

The experimental platform for realization of a *φ*-bit is composed of aluminum rods ½ inch in diameter and length on the order of two feet as elastic waveguides (6061 aluminum with certification: McMaster-Carr 1615T172). The waveguide is sandwiched along its length between heavy steel plates. The plates are 0.9525 cm thick by 5.08 cm wide and are forming a massive substrate. Four vises (with a clamping strength of 300 lbs, Home Depot: BV-HD60) are employed to apply a significant amount of pressure on the sandwich to establish a static friction force between the aluminum waveguide and the steel, high enough to ensure no slip conditions at their interface. The vises then contribute their mass to the substrate. The Hertzian contact between the rod and the steel substrate serves as elastic coupling between the two subsystems. The large mass difference between the rod and the substrate provides the limiting condition for the rod waveguide to achieve the behavior of a *φ*-bit.

We use two contact transducers ((Fingertip case style with 0.25 inch element diameter: Olympus V133-RM) attached to each end of the rod. One transducer is used to horizontally excite the rod and the other transducer is placed at the other end of the rod to measure the output response. The transducer driving the rod at one end is connected to a BK Precision 4055B arbitrary function generator (APG) and also to a Tektronix MDO3024 oscilloscope to register the driving signal generated by the APG. The other transducer reads the response signal transmitted to the other end of the rod. That response signal is registered in the same oscilloscope. To drive the rods we use a *sinc* pulse with a scan in frequency between 1 kHz and 100 kHz in steps of 50 Hz. The driving and response signals, averaged over 512 time series, are collected in the oscilloscope and the Fast Fourier Transform (FFT) calculated to get the frequency spectra. The ratio between the FFT of the response and driving signal is then calculated. Averages for each frequency are taken after the data collection is finished. The APG and oscilloscope are connected to a computer, which controls the experiment and performs data processing by using an in-house developed and implemented algorithm in MatLab R2018b.

As a reference, the transmission spectra of free standing aluminum rods are measured by suspending them with two thin strings. Given that the force transducer are not glued to the sample (i.e., depending on the frequency and amplitude of the excitation it could lose contact with the rod) and that the measured response might be affected by the rod-transducer dynamic interaction during the measurement, different couplants and ways of attaching the transducers to the rod are used. For ultrasonic couplants we use Gel (Olympus D12 ultrasonic couplant), kukui oil, and honey. The transducers are placed in contact with the end of the rods either by using rubber bands wrapping around the rods and transducers (supersize bands from Walmart, 564755837) or by placing the transducers on either side of the rod using fixed cylindrical lens mounts (Thorlabs, CH1A). In the experiment, we have also minimized the static pre-compression in the rod.

## Supplementary information

Experimental demonstration of coherent superpositions in an ultrasonic pseudospin
