# Supplementary material for: Experimental demonstration of coherent superpositions in an ultrasonic pseudospin
Source: Sci Rep. 2019 Oct 2;9:14156. doi: 10.1038/s41598-019-50366-y (PMC6775046; doi:10.1038/s41598-019-50366-y)
Supplement: Supplementary file 1 — Experimental demonstration of coherent superpositions in an ultrasonic pseudospin [file 41598_2019_50366_MOESM1_ESM.docx]

**Supplementary Materials**

**Experimental demonstration of coherent superpositions in an ultrasonic pseudospin**

Lazaro Calderin^*,1^, M. Arif Hasan^1^, Neil G Jenkins^1^, Trevor Lata^1^, Pierre Lucas^1^, Keith Runge^1^ and Pierre A. Deymier^1^

^1^Department of Materials Science and Engineering

University of Arizona, Tucson, Arizona, 85721, USA

^*^To whom correspondence should be sent; E-mail: [lcalderin@email.arizona.edu](mailto:lcalderin@email.arizona.edu)

Description of the COMSOL finite element mesh


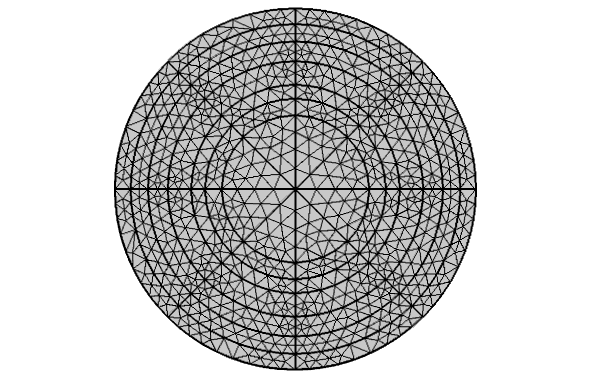

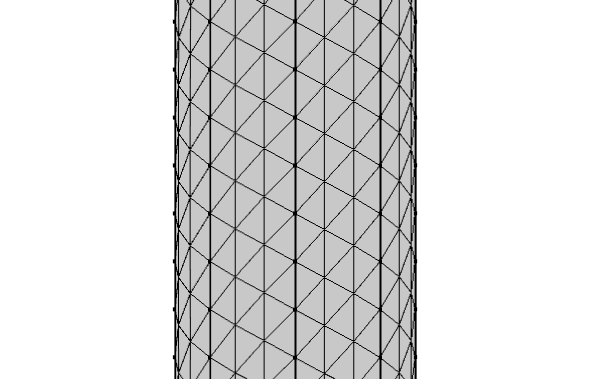


**Figure 1**: Finite elements grid (front and side views) used to calculate the eigenmodes of the free standing rods and $\varphi$-bits. Fixed boundaries imposed along the rod used to achieve $\varphi$-bit behavior are in marked in blue.
